# A qualitative inquiry on self-management decision making among rural African Americans with type 2 diabetes

**DOI:** 10.1093/geroni/igab046.2585

**Published:** 2021-12-17

**Authors:** Idethia Shevon Harvey

**Affiliations:** University of Missouri, Columbia, Missouri, United States

## Abstract

Living in a rural area has been recognized as a unique health disparity associated with higher rates of chronic disease. It is further compounded for those who are the most structurally vulnerable complicating access to care and negatively affecting health outcomes. Barriers to type 2 diabetes (T2DM) self-management remain a growing concern, particularly among minority communities living in underserved geographical areas. Much of the self-management research focused on compliance with medication regimens and modification of lifestyle choices. A less well-understood but arguably more critical aspect is the social factors in disease management decision-making. Purposive sampling was used to identify rural African Americans (n = 34). The mean age of participants was 65.9 years (SD = 12.3), and T2DM diagnosis was 15 years (SD = 12.4). The study utilized the consensual qualitative research methodology and the "Sort and Sift, Think and Shift" approach to identify themes. The participants reported an alternative way of integrating glucose monitoring through a "feedback loop" of body sensing. The longer they live with the condition (i.e., knowing my body), the more they can interpret whether they are hypoglycemic or hyperglycemic (i.e., deciphering the cues) to create and navigate their disease management strategy (i.e., body sensing). Self-management decision-making is a complex developmental process that includes disease trajectory and cultural and environmental factors. Findings from this study may provide a conceptual framework for ongoing inquiry and may provide insights to help T2DM educators and clinicians fully understand the complexity of long-term disease management among rural African Americans.

